# Pharmacokinetic analysis of vilobelimab, anaphylatoxin C5a and antidrug antibodies in PANAMO: a phase 3 study in critically ill,  invasively mechanically ventilated COVID-19 patients

**DOI:** 10.1186/s40635-023-00520-8

**Published:** 2023-06-19

**Authors:** Endry H. T. Lim, Alexander P. J. Vlaar, Sanne de Bruin, Simon Rückinger, Claus Thielert, Maria Habel, Renfeng Guo, Bruce P. Burnett, James Dickinson, Matthijs C. Brouwer, Niels C. Riedemann, Diederik van de Beek, the PANAMO study group, Martin Witzenrath, Pieter van Paassen, Leo M. A. Heunks, Bruno Mourvillier, Matthijs C. Brouwer, Pieter R. Tuinman, José Francisco K. Saraiva, Gernot Marx, Suzana M. Lobo, Rodrigo Boldo, Jesus A. Simon-Campos, Alexander D. Cornet, Anastasia Grebenyuk, Johannes M. Engelbrecht, Murimisi Mukansi, Philippe G. Jorens, Robert Zerbib, Korinna Pilz, Niels C. Riedemann, Pierre Bulpa, Fabio S. Taccone, Greet Hermans, Marc Diltoer, Michael Piagnerelli, Nikolaas De Neve, Antonio T. Freire, Felipe D. Pizzol, Anna Karolina Marinho, Victor H. Sato, Clovis Arns da Cunha, Mathilde Neuville, Jean Dellamonica, Djillali Annane, Antoine Roquilly, Jean Luc Diehl, Francis Schneider, Jean Paul Mira, Jean Baptiste Lascarrou, Luc Desmedt, Claire Dupuis, Carole Schwebel, Guillaume Thiéry, Matthias Gründling, Marc Berger, Tobias Welte, Michael Bauer, Ulrich Jaschinski, Klaus Matschke, Roberto Mercado-Longoria, Belinda Gomez Quintana, Jorge Alberto Zamudio-Lerma, Juan Moreno Hoyos Abril, Angel Aleman Marquez, Peter Pickkers, Luuk Otterspoor, Luis Hercilla Vásquez, Carlos Rafael Seas Ramos, Alejandro Peña Villalobos, Gonzalo Gianella Malca, Victoria Chávez, Victor Filimonov, Vladimir Kulabukhov, Pinak Acharya, Sjoerd A. M. E. G. Timmermans, Matthias H. Busch, Floor L. F. van Baarle, Rutger Koning, Liora ter Horst, Nora Chekrouni, Thijs M. van Soest, Marleen A. Slim, Lonneke A. van Vught, Rombout B. E. van Amstel, Sabine E. Olie, Ingeborg E. van Zeggeren, Marcel C. G. van de Poll, Dorothee Neukirchen

**Affiliations:** 1 Department of Intensive Care Medicine, Amsterdam UMC Location University of Amsterdam, AMC Room C3-421, Meibergdreef 9, 1105 AZ Amsterdam, The Netherlands; 2 Department of Neurology, Amsterdam UMC Location University of Amsterdam, Meibergdreef 9, Amsterdam, The Netherlands; 3 Metronomia Clinical Research GmbH, Munich, Germany; 4 InflaRx, Munich, Germany; 5 InflaRx, Jena, Germany; 6 InflaRx Pharmaceuticals Inc, Ann Arbor, MI USA

**Keywords:** PK, Pharmacokinetic, C5a, Complement, Vilobelimab, ADA, Antidrug antibodies, SARS-CoV-2, COVID-19, RCT

## Abstract

**Background:**

Vilobelimab, a complement 5a (C5a)-specific monoclonal antibody, reduced mortality in critically ill COVID-19 patients in a phase 3 multicentre, randomized, double-blind, placebo-controlled study. As part of the study, vilobelimab concentrations and C5a levels as well as antidrug antibodies (ADAs) to vilobelimab were analysed.

**Results:**

From Oct 1, 2020 to Oct 4, 2021, 368 invasively mechanically ventilated COVID-19 patients were randomized: 177 patients were randomly assigned to receive vilobelimab while 191 patients received placebo. Pharmacokinetic sampling was only performed at sites in Western Europe. Blood samples for vilobelimab measurements were available for 93 of 177 (53%) patients in the vilobelimab group and 99 of 191 (52%) patients in the placebo group. On day 8, after three infusions, mean vilobelimab (trough) concentrations ranged from 21,799.3 to 302,972.1 ng/mL (geometric mean 137,881.3 ng/mL). Blood samples for C5a measurements were available for 94 of 177 (53%) patients in the vilobelimab group and 99 of 191 (52%) patients in the placebo group. At screening, C5a levels were highly elevated and comparable between groups. In the vilobelimab group, median C5a levels were 118.3 ng/mL [IQR 71.2–168.2 ng/mL] and in the placebo group, median C5a levels were 104.6 ng/mL [IQR 77.5–156.6 ng/mL]. By day 8, median C5a levels were reduced by 87% in the vilobelimab group (median 14.5 ng/mL [IQR 9.5–21.0 ng/mL], p < 0.001) versus an 11% increase in the placebo group (median 119.2 ng/mL [IQR 85.9–152.1 ng/mL]). Beyond day 8, though plasma sampling was sparse, C5a levels did not reach screening levels in the vilobelimab group while C5a levels remained elevated in the placebo group. Treatment-emergent ADAs were observed in one patient in the vilobelimab group at hospital discharge on day 40 and in one patient in the placebo group at hospital discharge on day 25.

**Conclusions:**

This analysis shows that vilobelimab efficiently inhibits C5a in critically ill COVID-19 patients. There was no evidence of immunogenicity associated with vilobelimab treatment.

*Trial* *registration* ClinicalTrials.gov, NCT04333420. Registered 3 April 2020, https://clinicaltrials.gov/ct2/show/NCT04333420

**Supplementary Information:**

The online version contains supplementary material available at 10.1186/s40635-023-00520-8.

## Background

A randomised, double-blind, placebo-controlled, multicentre phase 3 trial (PANAMO, NCT04333420) showed that in addition to standard of care (SOC), vilobelimab (formerly IFX-1), a monoclonal antibody which specifically binds complement 5a (C5a), improved survival of invasively mechanically ventilated patients with coronavirus disease 2019 (COVID-19) [1]. Vilobelimab, in addition to SOC, reduced all-cause mortality at day 28 from 42 to 32% and protected against renal replacement therapy [1]. C5a is a potent anaphylatoxin attracting inflammatory cells to the site of infection, leading to tissue damage [2]. Increased activation of the complement system, in particular C5a, is associated with poor disease outcomes [3–6]. Previous research suggests that C5a inhibition decreases the inflammatory response and hypercoagulability in severe COVID-19 patients [7].

Vilobelimab is a chimeric monoclonal IgG4 antibody that binds with high affinity to the soluble form of human complement split factor C5a [8]. Experimental lung injury and influenza models have shown that the pharmacodynamic (PD) effect of vilobelimab blocking C5a resulted in a reduction of granulocyte activation, neutrophil chemotaxis to the site of tissue damage and a reduction in systemic inflammatory responses [9, 10]. Earlier, in a substudy of an exploratory, open-label phase 2 trial as part of PANAMO, we showed that vilobelimab suppressed C5a levels in 10 severely ill COVID-19 patients after a single infusion, as compared to 12 controls, which was maintained for at least eight days [8]. As part of the phase 3 PANAMO trial, drug concentrations for vilobelimab were analysed alongside C5a levels and the occurrence of antidrug antibodies (ADAs) to vilobelimab in invasively mechanically ventilated patients with COVID-19.

## Methods

From October 1, 2020 to October 4, 2021, 369 critically ill COVID-19 patients were included in the phase 3 PANAMO trial in 46 hospitals in Europe, Africa and North- and South-America. The PANAMO trial was a multicentre, double-blind, randomised, placebo-controlled, phase 3 trial [1]. Inclusion criteria were an age of 18 years or older, invasive mechanical ventilation within 48 h before the first infusion of study medication, a PaO_2_/FiO_2_ (P/F ratio) of 60–200 mm Hg and a confirmed severe acute respiratory syndrome coronavirus 2 (SARS-CoV-2) infection in the past 14 days. The complete exclusion criteria can be found in the original article of the phase 3 study results [1].

Overall, 369 patients were enrolled into the trial. One patient in the vilobelimab group was randomized in error and was thus excluded. Therefore, 177 patients were randomly assigned to receive vilobelimab and SOC and 191 patients were randomly assigned to receive a matching placebo and SOC. Vilobelimab was administered intravenously with six, 800 mg dosages on days 1, 2, 4, 8, 15 and 22, while at the intensive care unit (ICU) and during hospital stay. The first infusion had to be administered within 48 h after randomization.

Pharmacokinetic (PK) sampling was only performed at sites in Western Europe. Blood samples for vilobelimab, C5a and ADAs to vilobelimab were taken before infusion of study medication at screening, *i.e*., before infusion of study medication, at day 8 and at hospital discharge. Patients with at least one study infusion and an evaluable C5a assessment at either baseline or day 8 were included in this analysis. The vilobelimab (trough) plasma drug and C5a levels were analysed by menal GmbH, a laboratory contracted by InflaRx GmbH, the study sponsor, utilizing an enzyme-linked immunosorbent assay (ELISA). The ELISAs were developed by InflaRx GmbH and validated by menal GmbH. A summary of validation parameters of the vilobelimab and C5a ELISA is added to the supplementary information (Additional file 1: Table S1). ADA measurement was performed in serum using an electrochemiluminescence (ECL), Meso Scale Discovery (MSD) based bridging assay (Eurofins BioPharma Product Testing Munich GmbH). In the bridging assay, vilobelimab was applied as capture and detection reagent. Polyclonal rabbit anti-vilobelimab antibodies served as positive reference material. The test system was validated for the detection and characterization of potential ADA directed against vilobelimab in human serum samples using the multi-tiered approach: (i) screening assay; (ii) confirmatory assay and (iii) titration assay to characterize the amplitude of the immune response. In the screening assay, all study samples are screened for ADA positive signals with an acceptance of 5% false positive results. All screened positive samples are measured in the ADA confirmatory assay with an acceptance of 1% false positive results. All confirmed positive samples are characterized in the ADA titration assay for semi-quantification with an acceptance of 0.1% false positive results.

Actual PK sampling times were determined and the plasma concentration of vilobelimab was assessed by time point. After unblinding, the sponsor reviewed sampling dates and vilobelimab concentrations and flagged samples in the following two cases: (i) implausible vilobelimab/C5a concentration; (ii) timing of sample not pre-dose or other issue with timing. Data points were only omitted from the analysis in these two cases. Data are expressed by descriptive statistics, including mean with standard deviation (SD), geometric mean and medians with interquartile range [IQR]. C5a levels and their changes from baseline at day 8 were compared between both groups applying the Wilcoxon rank-sum test. Correlations between continuous variables were assessed with Spearman’s rank correlation coefficient. A p-value below 0.05 was considered as statistically significant. All statistical analyses were performed in SAS (version 9.4).

## Results

Of all patients in the PK analysis, 21 of 96 (22%) patients in the vilobelimab group and 37 of 101 (37%) patients in the placebo group had died at day 28. At day 60, 28 of 96 (29%) patients in the vilobelimab group and 43 of 101 (43%) patients in the placebo group had died. Screening blood samples for vilobelimab measurement were available for 93 of 177 (53%) patients in the vilobelimab group and 99 of 191 (52%) patients in the placebo group. For some patients, samples were only available at screening or day 8. Samples were taken exclusively from Western European patients: in the Netherlands, 129 (66%) patients; in France, 33 (17%) patients; in Germany, 21 (11%) patients; and in Belgium, 14 (7%) patients (Table 1).

**Table 1:** Baseline characteristics

	Total (n = 197)	Vilobelimab (n = 96)	Placebo (n = 101)
Age (years)			
Mean	61.4 (12.3)	60.4 (12.8)	62.3 (11.9)
Min–max	23–81	23–81	23–81
Median	64.0 [54.0–71.0]	64.0 [53.0–71.0]	64.0 [55.0–71.0]
Sex			
Male	144 (73.1%)	73 (76.0%)	71 (70.3%)
Female	53 (26.9%)	23 (24.0%)	30 (29.7%)
Country			
Belgium	14 (7.1%)	7 (7.3%)	7 (6.9%)
Germany	21 (10.7%)	10 (10.4%)	11 (10.9%)
France	33 (16.8%)	16 (16.7%)	17 (16.8%)
Netherlands	129 (65.5%)	63 (65.6%)	66 (65.3%)
Race			
White	129 (65.5%)	66 (68.8%)	63 (62.4%)
Asian	9 (4.6%)	4 (4.2%)	5 (5.0%)
Black or African American	9 (4.6%)	2 (2.1%)	7 (6.9%)
Other	21 (10.7%)	11 (11.5%)	10 (9.9%)
Not reported	29 (14.7%)	13 (13.5%)	16 (15.8)
BMI (kg/m^2^)			
Mean	30.5 (5.7)	30.8 (5.3)	30.1 (6.1)
Min–max	18–55	22–46	18–55
Median	29.5 [26.6–32.8]	30.3 [27.4–32.7]	28.8 [26.1–32.9]
Estimated glomerular filtration rate			
< 60 mL/min/1.73m^2^	61 (31.0%)	28 (29.2)	33 (32.7)
≥ 60 mL/min/1.73m^2^	136 (69.0%)	68 (70.8%)	68 (67.3%)
Comorbidities			
Hypertension	95 (48.2%)	42 (43.8%)	53 (52.5%)
Diabetes	73 (37.1%)	32 (33.3%)	41 (40.6%)
Coronary heart disease	23 (11.7%)	11 (11.5%)	12 (11.9%)
Chronic obstructive pulmonary disease	5 (2.5%)	4 (4.2%)	1 (1.0%)
Carcinoma	3 (1.5%)	1 (1.0%)	2 (2.0%)
Chronic kidney disease	19 (9.6%)	7 (7.3%)	12 (11.9%)
Obesity	62 (31.5%)	29 (30.2%)	33 (32.7%)
Acute respiratory distress syndrome			
Mild (PaO_2_/FiO_2_ 200–300 mm Hg)^a^	2 (1.0%)	1 (1.0%)	1 (1.0%)
Moderate (PaO_2_/FiO_2_ 100–200 mm Hg)	145 (73.6%)	72 (75.0%)	73 (72.3%)
Severe (PaO_2_/FiO_2_ ≤ 100 mm Hg)	50 (25.4%)	23 (24.0%)	27 (26.7%)
8-point WHO COVID-19 ordinal scale			
6 (intubation and mechanical ventilation)	47 (23.9%)	29 (30.2%)	18 (17.8%)
7 (ventilation plus organ support)	150 (76.1%)	67 (69.8%)	83 (82.2%)
Time between onset of first COVID-19 symptoms and randomization (days)			
Mean	10.1 (5.7)	10.4 (5.4)	9.9 (6.0)
Min–max	0–31	0–31	0–29
Median	10.0 [6.0–14.0]	11.0 [6.0–14.0]	10.0 [5.0–14.0]
Time between hospital admission and randomization (days)			
Mean	4.3 (3.9)	3.9 (3.0)	4.7 (4.6)
Min–max	0–27	0–19	0–27
Median	4.0 [2.0–5.0]	3.5 [2.0–5.0]	4.0 [2.0–6.0]
Time between ICU admission and randomization (days)			
Mean	2.5 (3.1)	2.2 (1.8)	2.9 (3.9)
Min–max	0–22	0–11	0–22
Median	2.0 [1.0–3.0]	2.0 [1.0–3.0]	1.0 [1.0–3.0]
Time between intubation onset and first IMP administration (hours)			
Mean	22.2 (14.4)	23.5 (15.1)	20.8 (13.7)
Min–max	0–52	0–52	3–51
Median	23.0 [11.2–37.6]	24.2 [11.5–40.8]	22.8 [10.6–33.6]

Data are n (%), mean (SD), median [IQR]

^a^Two patients were included with a PaO_2_/FiO_2_ ratio > 200 mm Hg, despite the inclusion criterion of 60–200 mm Hg

BMI, Body mass index; COVID-19, Coronavirus disease 2019; ICU, Intensive care unit; IMP, Investigational medicinal product; IQR, Interquartile range; Max, Maximum; Min, Minimum; PaO_2_/FiO_2_, Partial pressure of oxygen in arterial blood/Fraction of inspired oxygen ratio; SD, Standard deviation; WHO, World Health Organization

Screening blood samples for C5a measurement were available for 94 of 177 (53%) patients in the vilobelimab group and 99 of 191 (52%) patients in the placebo group. Samples were not always available at both screening and day 8. Baseline characteristics were comparable between treatment groups (Table 1), although a slightly larger proportion in the placebo group received mechanical ventilation and additional organ support as compared to the vilobelimab group. The median age of patients was 64 years [IQR 54–71] and 144 (73%) patients were male. The most common comorbidities were hypertension, diabetes, and obesity (Table 1). Most patients had moderate acute respiratory distress syndrome (ARDS), classified as a P/F ratio between 100- and 200 mm Hg.

**Fig. 1:**
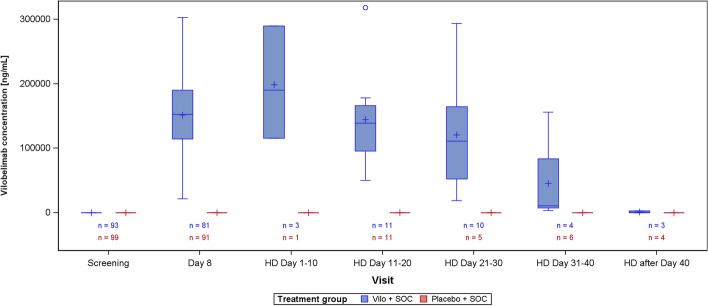
Vilobelimab drug concentration. 5 value(s) from vilobelimab patients and 1 value(s) from placebo patients have been excluded from this figure due to incorrect timing or implausible values. Values below the lower limit of quantification are set to zero. Values above the upper limit of quantification are set to the upper limit of quantification. Box plot: lower line of box = 1st quartile, line inside box = median, upper line of box = 3rd quartile, +  = mean, lower/upper whisker = minimum/maximum value below/above lower/upper line of box + 1.5 * (3rd quartile–1st quartile), circle = values below/above whiskers. *HD*  Hospital discharge; *SOC*  Standard of care; *Vilo*  Vilobelimab

**Fig. 2:**
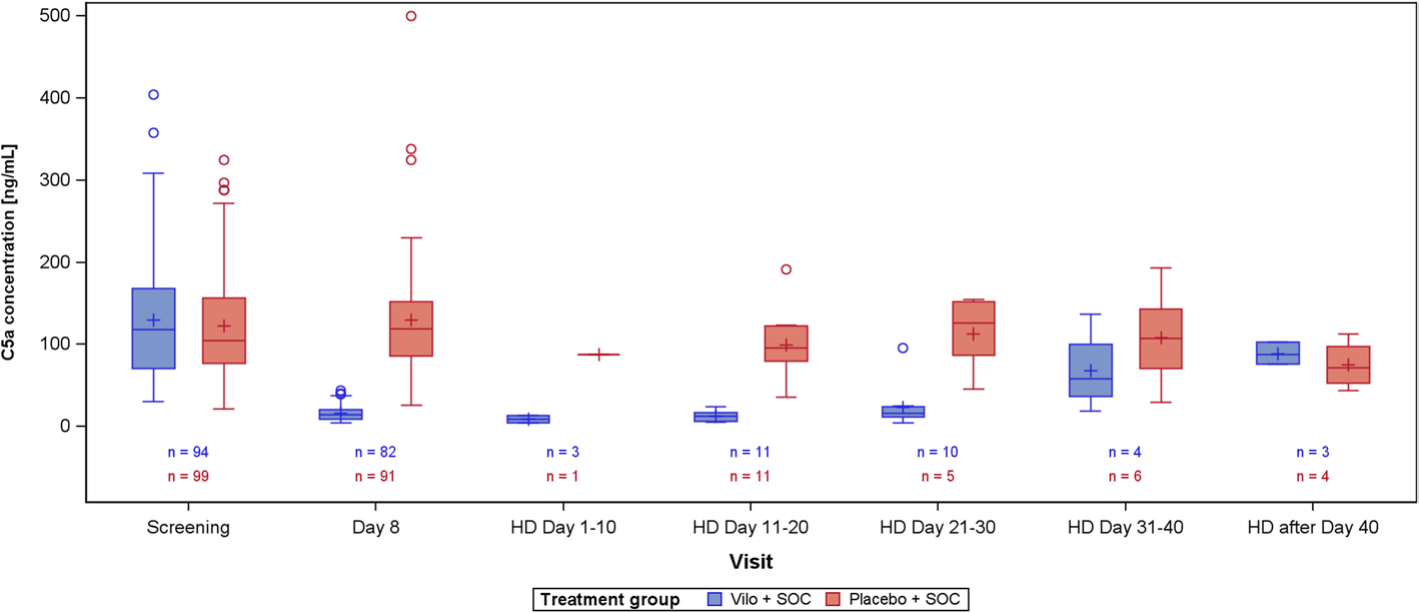
C5a concentration. 5 value(s) from vilobelimab patients and 1 value(s) from placebo patients have been excluded from this figure due to incorrect timing or implausible values. Values below the lower limit of quantification are set to zero. Values above the upper limit of quantification are set to the upper limit of quantification. Box plot: lower line of box = 1st quartile, line inside box = median, upper line of box = 3rd quartile, +  = mean, lower/upper whisker = minimum/maximum value below/above lower/upper line of box + 1.5 * (3rd quartile–1st quartile), circle = values below/above whiskers. *HD*  Hospital discharge; *SOC* Standard of care, *Vilo* Vilobelimab

Vilobelimab levels were not measurable in either treatment group at screening, but on day 8, after three infusions, mean vilobelimab (trough) concentrations were 21,799.3 to 302,972.1 ng/mL (geometric mean 137,881.3 ng/mL) (Fig. 1). At screening, C5a concentrations were highly elevated and comparable between groups (Fig. 2). In the vilobelimab group, median C5a levels were 118.3 ng/mL [IQR 71.2–168.2 ng/mL] and in the placebo group, median C5a levels were 104.6 ng/mL [IQR 77.5–156.6 ng/mL]. At baseline, there was no association between C5a levels of all patients and P/F ratio, ARDS severity, WHO ordinal scale, mortality, kidney function measured in estimated glomerular filtration rate, and thrombotic complications. Correlations at day 8 between C5a and leukocytes were weak in the vilobelimab group (r_s_ 0.26) and negligible in the placebo group (r_s_ 0), and correlations between C5a and CRP at day 8 were moderate in the vilobelimab group (r_s_ 0.56) and weak in the placebo group (r_s_ 0.32).

By day 8, median C5a levels were reduced by 87% in the vilobelimab group (median 14.5 ng/mL [IQR 9.5–21.0 ng/mL]) versus a 11% increase in the placebo group (median 119.2 ng/mL [IQR 85.9–152.1 ng/mL]), resulting in a statistically significant difference between the two groups (p < 0.001). Beyond day 8, although plasma sampling was sparse, C5a levels remained elevated in the placebo group (Fig. 2**)**. In the vilobelimab group, C5a levels began to rise after end of treatment for patients discharged from day 30, but were still lower than levels recorded at baseline.

**Fig. 3:**
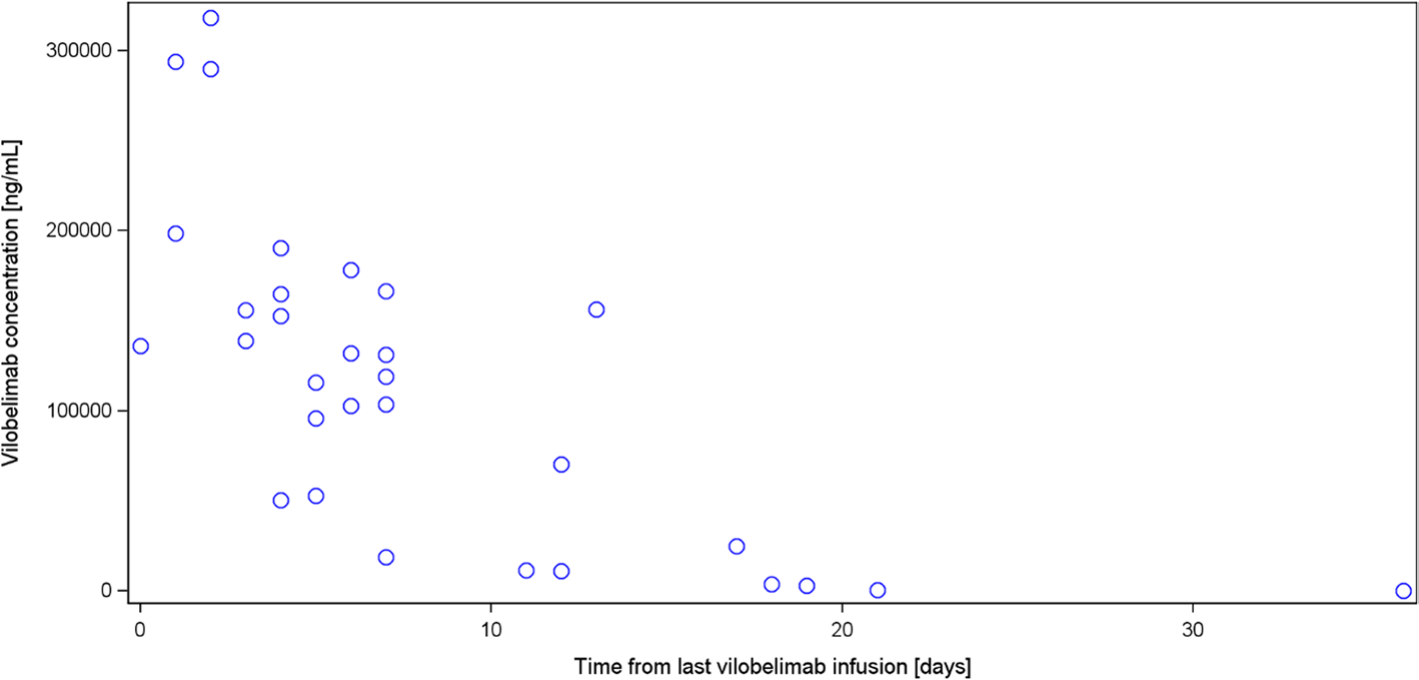
Scatterplot of vilobelimab concentration at hospital discharge by time from last vilobelimab infusion

**Fig. 4:**
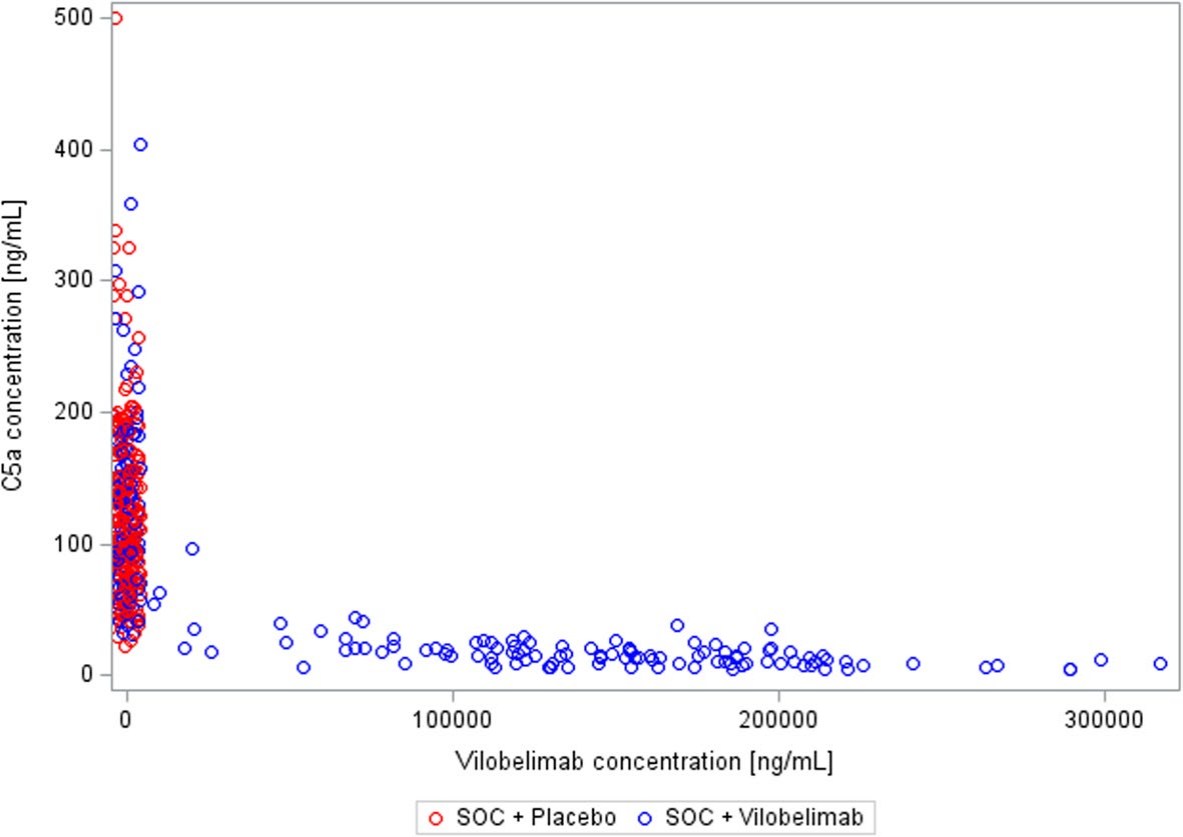
Scatterplot of Vilobelimab and C5a concentration (all available data, irrespective of visit). 9 value(s) from vilobelimab patients and 3 value(s) from placebo patients have been excluded from this figure due to incorrect timing or implausible values. Values below the lower limit of quantification are set to zero. Values above the upper limit of quantification are set to the upper limit of quantification. *SOC* Standard of care; *Vilo*  Vilobelimab

Vilobelimab concentration at hospital discharge by time from last vilobelimab infusion is illustrated in Fig. 3. The PK data of vilobelimab and C5a is shown in Table 2. The relationship between vilobelimab and C5a levels for both groups was also assessed (Fig. 4). Treatment-emergent ADAs were observed in one patient in the vilobelimab group at day 40 on hospital discharge and in one patient in the placebo group at day 25 on hospital discharge.

**Table 2:** Pharmacokinetic data of vilobelimab and C5a [ng/mL]

Visit statistic	Vilobelimab concentration		C5a concentration	
	Absolute value		Absolute value	
	Vilo + SOC (N = 96)	Placebo + SOC (N = 101)	Vilo + SOC (N = 96)	Placebo + SOC (N = 101)
Screening				
n	93	99	94	99
Mean (SD)	0.00 (0.00)	0.00 (0.00)	130.25 (71.45)	123.15 (65.53)
Geom. Mean	n.a	n.a	112.83	106.95
Day 8				
n	81	91	82	91
Mean (SD)	151,236.35 (56,782.86)	0.00 (0.00)	16.80 (9.15)	129.81 (67.59)
Geom. Mean	137,881.29	n.a	14.54	116.03
HD Day 1–10				
n	3	1	3	1
Mean (SD)	198,506.84 (87,412.05)	0.00 ()	9.06 (4.32)	87.70 ()
Geom. Mean	185,361.06	n.a	8.29	87.70
HD Day 11–20				
n	11	11	11	11
Mean (SD)	144,856.82 (69,976.35)	0.00 (0.00)	13.13 (6.30)	99.93 (39.06)
Geom. Mean	130,591.95	n.a	11.74	93.00
HD Day 21–30				
n	10	5	10	5
Mean (SD)	121,030.00 (83,574.67)	0.00 (0.00)	23.82 (26.19)	113.20 (46.22)
Geom. Mean	91,085.66	n.a	17.23	103.59
HD Day 31–40				
n	4	6	4	6
Mean (SD)	45,638.49 (73,828.83)	0.00 (0.00)	68.37 (49.74)	108.75 (56.91)
Geom. Mean	16,579.17	n.a	54.65	93.57
HD after Day 40				
n	3	4	3	4
Mean (SD)	1235.72 (1552.04)	0.00 (0.00)	88.99 (13.82)	75.27 (29.67)
Geom. Mean	493.34	n.a	88.28	70.89

5 value(s) from vilobelimab patients and 1 value(s) from placebo patients have been excluded from this summary table due to incorrect timing or implausible values. Values below the lower limit of quantification are set to zero. Values above the upper limit of quantification are set to the upper limit of quantification

*SD* Standard deviation, *Geom*. *Mean* Geometric mean, *SOC* Standard of care, *Vilo* Vilobelimab

## Discussion

The PK relationship observed in the phase 3 PANAMO trial confirms that vilobelimab efficiently removes C5a from circulation in critically ill, COVID-19 patients. Our results confirm that C5a blood levels are highly elevated in critically ill, COVID-19 patients [8]. After three infusions of vilobelimab by day 8, C5a was significantly suppressed in the vilobelimab group compared to the placebo group, where C5a levels slightly increased. The PK results also show that C5a levels remained high throughout the study in the placebo group and began to rise in the vilobelimab group to sub-baseline levels only after end-of-treatment for patients discharged after day 30. Vilobelimab drug levels were sufficient for complete pharmacological action in suppressing C5a using this dosing regimen, which was based on an observed PK/PD relationship in the phase 2 part of PANAMO that demonstrated the same efficient inhibition of C5a [8].

Administration of monoclonal antibodies, like any other biological agent, can lead to development of ADAs, induced by a humoral response when recognized by the immune system [11, 12]. This can lead to loss of drug activity via several mechanisms, such as blockade of the drug target binding by neutralizing antibodies, or by increased clearance of the ADA-drug complex, which leads to reduced drug plasma concentrations [12, 13]. Also, the generation of ADAs can induce toxicities due to an immune response to the ADA-drug complex, such as infusion-related reactions [12, 14]. In our study, treatment-emergent ADAs were observed in one patient in the vilobelimab group and one patient in the placebo group, however neither case appeared to influence the safety of vilobelimab. No adverse events related to immunogenicity or related safety findings were observed in these patients. Thus, there was no evidence of immunogenicity associated with this dosing regimen of vilobelimab, based on the absence of any ADAs in almost all patients, the maintenance of vilobelimab drug concentrations and a lack of safety signals. The ADAs found in the patient receiving placebo might have been the result of a false positive readout due to pre-existing autoantibodies or related to unspecified interference with the assay setup, such as high C5a levels or interference with other drugs [15]. At baseline, we found no association between C5a levels and disease characteristics or WHO COVID-19 ordinal scale scoring.

In the phase 3 PANAMO trial, vilobelimab significantly improved survival of critically ill, COVID-19 patients and protected against renal replacement therapy [1]. In an exploratory substudy of the phase 2 PANAMO trial, we observed that ADAMTS13, which cleaves von Willebrand factor multimers, remained stable in the vilobelimab group whereas ADAMTS13 levels decreased significantly in the control group, most likely due to consumption [7]. This might suggest that inhibition of C5a can reduce endotheliopathy caused by COVID-19 and thereby protects against thrombotic complications in severely ill COVID-19 patients [7, 16]. Vilobelimab also appeared to reduce levels of the proinflammatory chemokine interleukin-8 (IL-8) in the phase 2 PANAMO trial [7], which plays a significant role in the pathogenesis of ARDS and likely COVID-19 as well [7, 17, 18].

There are several studies investigating inhibition of the complement system on different targets in COVID-19 [5]. However, specifically inhibiting C5a instead of inhibiting upstream of C5a has several advantages. First, C5a seems to be a key factor involved in the pathogenesis of patients with severe COVID-19 rather than other complement factors [1, 3, 5, 19]. Second, in order to inhibit C5a substantially, specific inhibition of C5a is required [5, 19, 20]. Namely, C5a can be generated outside the common complement pathways by enzymes such as trypsin and thrombin [1, 8, 20–22], which is important given frequent thrombotic complications in COVID-19 [16]. The C5 inhibitor eculizumab was shown not to reduce C5a levels in severe COVID-19 patients [23]. Third, specifically inhibiting C5a does not affect cleavage of C5 into C5b, part of the membrane attack complex (MAC) [9, 20, 24], which plays an important role in host defence through cell lysis [25]. Targeting the complement system upstream of C5a inevitably and negatively affects formation of the MAC [1, 5, 8, 10, 19, 21]. C5 inhibition in COVID-19, for example, is associated with an increased risk of secondary infections [5, 26], whereas targeting C5a does not presumably due to the preservation of the MAC [1, 21].

Our study has a few limitations. First, we only performed PK sampling in Western Europe. This could have introduced selection bias. However, it is a representative sample and PK sampling was available for 197/368 (54%) of all patients included in the complete trial. This study includes the largest population showing the drug concentration relationship with C5a inhibition in critically ill COVID-19 patients to date. Second, we only have C5a measurements at screening, day 8 and hospital discharge. We did not draw samples at hospital or ICU admission, since we could only include patients after they were invasively mechanically ventilated and met all inclusion criteria. Nonetheless, the median time between hospital admission and randomization was only four days. Beyond day 8, plasma sampling was sparse. Yet, it was clear that C5a levels remained elevated in the placebo group throughout the study whereas, in the vilobelimab group, C5a levels began to rise only after end of treatment for patients discharged from day 30, but not to levels recorded at baseline. Third, no specific PD assessments were performed in this study looking at neutrophil activation or other inflammatory markers. However, based on known high levels of C5a and neutrophil activation in COVID-19 and the presence of neutrophil extracellular trap (NET)osis in the microvasculature [8, 27, 28], *i.e*., microthrombosis which contributes to mortality [28], significant improvement in survival of critically ill, invasively mechanically ventilated COVID-19 patients with vilobelimab are consistent with its presumed PD effect [1].

## Conclusions

This observed PK relationship shows that vilobelimab efficiently inhibits C5a in critically ill COVID-19 patients thus accounting for the clinical efficacy observed in the PANAMO study. There was no evidence of immunogenicity associated with this dosing regimen of vilobelimab, based on the absence of any ADAs in almost all patients and the maintenance of vilobelimab drug concentrations.

## Supplementary Information

**Additional file 1: Table S1. **Summary of validation parameters of vilobelimab and C5a ELISA.

## Data Availability

Data will be shared according to applicable regulatory requirements.

## Abbreviations

ADAs: Antidrug antibodies
ADAMTS13: A disintegrin and metalloproteinase with a thrombospondin type 1 motif, member 13
ARDS: Acute respiratory distress syndrome
C5: Complement factor 5
C5a: Complement factor 5a
COVID-19: Coronavirus disease 2019
CV: Coefficient of variation
ECL: Electrochemiluminescence
ELISA: Enzyme-linked immunosorbent assay
FiO_2_: Fraction of inspired oxygen
HD: Hospital discharge
ICU: Intensive care unit
IL-8: Interleukin-8
IgG: Immunoglobulin G
IMP: Investigational medicinal product
IRB: Institutional review board
IQR: Interquartile range
MAC: Membrane attack complex
Max: Maximum
Min: Minimum
MSD: Meso Scale Discovery
NET: Neutrophil extracellular trap
P/F: PaO_2_/FiO_2_
PaO_2_: Partial pressure of oxygen in arterial blood
PD: Pharmacodynamic
PK: Pharmacokinetic
Q1: First quartile
Q3: Third quartile
SARS-CoV-2: Severe acute respiratory syndrome coronavirus 2
SD: Standard deviation
SOC: Standard of care
Vilo: Vilobelimab
WHO: World Health Organization
