# Supplementary material for: Pharmacokinetic analysis of vilobelimab, anaphylatoxin C5a and antidrug antibodies in PANAMO: a phase 3 study in critically ill, invasively mechanically ventilated COVID-19 patients
Source: Intensive Care Med Exp. 2023 Jun 19;11:37. doi: 10.1186/s40635-023-00520-8 (PMC10277268; doi:10.1186/s40635-023-00520-8)
Supplement: Supplementary file 1 — Additional file 1: Table S1. Summary of validation parameters of vilobelimab and C5a ELISA. [file 40635_2023_520_MOESM1_ESM.docx]

**Supplementary Information**

**Table S1** Summary of validation parameters of vilobelimab and C5a ELISA

menal GmbH, vilobelimab ELISA

| **Items** | **Results** |
| --- | --- |
| **Methodology** | ELISA assay |
| **Biological matrix** | human plasma |
| **Anticoagulant (if applicable)** | Sodium Citrate |
| **Calibration curve range** | 12.5 – 225 ng/mL |
| **Analyte of interest** | vilobelimab |
| **Inter-run accuracy** | 91 – 108% |
| **Inter-run precision** | 9 – 23% |
| **Dilution Linearity** | Dilution linearity/parallelism is given for dilution factor 4 – 64,000 |

menal GmbH, C5a ELISA

| **Items** | **Results** |
| --- | --- |
| **Methodology** | ELISA assay |
| **Biological matrix** | human plasma |
| **Anticoagulant (if applicable)** | Sodium Citrate |
| **Calibration curve range** | 0.0625 – 2.00 ng/mL |
| **Analyte of interest** | C5a |
| **Inter-run accuracy** | 94 – 99% |
| **Inter-run precision** | 4 – 8% |
| **Dilution Linearity** | Dilution linearity/parallelism is given for dilution factor 40 - 250 |
| **C5a reference values in healthy donors** | 37.3 – 49.8 ng/mL (n = 40) |
